# Changes in Prevalence and Socioeconomic Factors of Psychiatric Disability among Children in China from 1987–2006: A Population Based Survey

**DOI:** 10.3390/ijerph14030279

**Published:** 2017-03-09

**Authors:** Zhenjie Wang, Xiaoying Zheng, Lei Zhang, Gong Chen

**Affiliations:** 1 Institute of Population Research/WHO Collaborating Center on Reproductive Health and Population Science, Peking University, Beijing 100871, China; wangzhenjie1981@aliyun.com (Z.W.); zhang.lei@pku.edu.cn (L.Z.); chengong@pku.edu.cn (G.C.); 2 Laboratory of Neuroscience and Mental Health, Peking University, No.5 Yiheyuan Road, Haidian District, Beijing 100871, China

**Keywords:** psychiatric, disability, socioeconomic, children, China

## Abstract

The prevalence and risk factors associated with psychiatric disability among Chinese children under 14 years of age has long been of interest. Data used in the study included two nationally representative population-based surveys from the first and second China National Sample Surveys on Disability, conducted in 1987 and 2006. Both surveys used multistage, stratified random cluster sampling, with probability proportion to size, to derive nationally representative samples. Age-standardized point prevalence of mental disability was estimated through direct standardization using the 2000 census-derived Chinese population as the standard. Associations between psychiatric disability in children and possible risk factors were examined by logistic regression. Age-standardized point prevalence of psychiatric disability in children increased sharply from 0.18% to 1.11% in the 20 years between surveys. In the logistic regression analysis, the children’s age and household size presented inverse associations with psychiatric disability in both surveys, although these associations were not all significant in 1987. Residential area, minority group status and gender of children were consistently associated with psychiatric disability in both surveys. To face the challenge of rising prevalence rates of psychiatric disability among children in China, the government should adopt more vigorous strategies to prevent it, especially for minority ethnicity children and those living in rural areas.

## 1. Introduction

Children with disabilities are among the most vulnerable members of any society [1]. The WHO Global Burden of Disease report estimates the number of children aged 0–14 years with “moderate or severe disabilities” at 93 million (5.1% of the global child population), with 13 million (0.7%) children experiencing severe difficulties [2]. UNICEF has estimated the number of children with disabilities under age 18 years at around 150 million [3], but the WHO has estimated that nearly 200 million children worldwide have a disability [4], and a disproportionate number of these children live in developing countries [5,6]. The prevalence of children with disabilities varies substantially, depending on the definition and measure of disability [7]. The worldwide prevalence rates for child and adolescent mental disorders are around 10% to 20% [8], and the prevalence of mental disorders in developing countries ranges from 1.8% to 17.7% [9].

Half of all lifetime mental disorders begin before the age of 14 years [10,11]. An important birth cohort study suggested that half its subjects had a psychiatric diagnosis by 15 years of age [12], and childhood psychotic symptoms have been shown to be associated with the development of schizophrenia in adulthood [13]. Moreover, children in developing countries are exposed to multiple risks including poverty, malnutrition, and poor health and home environments, which can impair cognitive, motor, and social-emotional development [14].

However, no studies have explored the prevalence of changes in psychiatric disability among Chinese children, particularly from 1987 to 2006. In the study reported here, we investigated the prevalence and associations between demographic factors and psychiatric disability in Chinese children aged less than 14 years, using data from two nationally representative population-based surveys [15,16].

## 2. Methods

### 2.1. Data Source

Data were derived from a nationally representative population-based survey, the China National Sample Survey on Disability conducted in 1987 and 2006. Both surveys used multistage, stratified random cluster sampling, with probability proportional to size, to derive nationally representative samples. Within each province, sampling strata were defined based on subordinate administrative areas, local geographical characteristics or local gross domestic product, where appropriate, to allow for anticipated regional variability. Within each stratum, a four-stage sampling strategy was followed involving four natural administrative units, and sampling was conducted with probability proportional to cluster size. The sampling interval used the most up-to-date population and address information from the Ministry of Civil Affairs and Public Security in Beijing. The protocol and questions of these surveys were reviewed by leading national and international experts, and the sampling scheme was reviewed by experts from the United Nations Statistics Division [15,16]. The sampling ratio was 1.50 per 1000 population for 1987 survey and 1.93 per 1000 population for 2006 survey [15,16]. Details of the study design and methods are described elsewhere [17]. The current study population comprised respondents aged 14 years or less from both surveys.

### 2.2. Ethics

These surveys were approved by the State Council (Guo Ban Fa No. 77 (1986); Guo Ban Fa No 73 (2004)) and the survey plan was formulated in accordance with the Statistics Law of the People’s Republic of China and the Law of the People’s Republic of China on the Protection of Disabled Persons. During the surveys, all respondents provided informed consent to participate in the surveys and clinical diagnosis.

### 2.3. Data Collection Procedures and Data Quality

Two pilot studies were conducted in different provinces before each survey, to collect accurate information about the suspected numbers of disabled children under age 6 years. Strict quality control measures were implemented at every step during the survey, from drafting the sampling frame through field sampling, from filling out the questionnaires to checking the returned forms, and from data input to checking the data quality [15,16]. During data collection, the investigation teams included a team leader, a deputy team leader, a deputy medical team leader, investigators, statisticians, and medical doctors with expertise in otolaryngology, ophthalmology, surgery/orthopedics, psychiatry, and pediatrics. Furthermore, a designated physician performed medical examinations and followed diagnostic manuals to make the final diagnosis, assess the severity of disability, if any, and to confirm its primary causes. Respondents with multiple positive diagnoses were examined by multiple specialists (a separate physician for each disability).

After the field investigations were concluded, the teams made home re-visits to conduct surveys in the quarters chosen for post-survey quality checks and calculate errors in the survey overall. The results of the quality checks showed that the omission rate of the resident population was 1.06 per 1000 persons in 1987 and 1.31 per 1000 persons in 2006; the omission rate of the disabled population was 1.16 per 1000 persons in 1987 and 1.12 per 1000 persons in 2006 [15,16].

### 2.4. Identification of People with Psychiatric Disability

In both 1987 and 2006, trained field interviewers used a structured questionnaire to inquire about psychiatric disability among children (Table 1 and Table 2). Those who responded “yes” to any of the corresponding questions were referred to different designated physi­cians for further disability screening and confirmation. Psychiatric disability cases were defined and classed by the expert committee of the China National Sample Survey on Disability based on the definition of psychiatric disability: “Psychiatric disability refers to mental disorders lasting more than one year, which manifest in cognitive, affective and behavior disorders that limit one’s daily life and restrict participation”; this was done for 1987 and 2006 [15,16]. The 1987 survey is based on the International Classification of Impairment, Disability and Handicap [18] and the 2006 survey is according to the International Classification of Functioning, Disability and Health [19] in design. Child psychiatric disorders were diagnosed by experienced psychiatry physicians according to the Chinese Classification of Mental Disease (CCMD) in 1987, which developed by referencing International Statistical Classification of Diseases, 9th Revision (ICD-9) [20,21] and the International Statistical Classification of Diseases, 10th Revision (ICD-10) in 2006 [22]. Although there were unavoidable differences between CCMD and ICD-10, these two classifications were comparable [22,23]. The classification, screening method, diagnosis method, and relevant disability scales were pretested in pilot studies, with good reliability, validity and comparable [15,16,23,24,25].

### 2.5. Study Variable Definition

We defined the status of psychiatric disability as binary (i.e., yes or no); the age group as early childhood (0–3 years), pre-school (4–5 years) and school age (6–14 years); gender as male or female; residential area as urban or rural; ethnicity as Han or other; and household size as 1–3, 4–6 and 7–9 persons/household. For final analysis, the current study population comprised 460,404 from the 1987 survey, including 78 psychiatric disability cases and 479,581 from the 2006 survey, including 525 psychiatric disability cases. Age-standardized point prevalence of psychiatric disability was estimated through direct standard­ization using the 2000 census-derived Chinese population as the standard [26]. The difference proportion was tested by chi-squared test or Fisher’s exact chi-squared test between the 1987 and 2006 surveys. A logistic regression model was used to calculate the adjusted odds ratios (OR) and 95% confidence interval (CI) of psychiatric disability for the variables. The results were declared statistically significant if the two-sided *p*-value was <0.05. Statistical analyses were performed using SAS 9.2 software (SAS Institute, Inc., Cary, NC, USA).

## 3. Results

Selected characteristics of children aged 0–14 years are summarized in Table 1. Overall, the age-standardized point prevalence of psychiatric disability increased from 0.18‰ in 1987 to 1.11‰ in 2006. Without taking epilepsy into consideration, the age-standardized point prevalence of psychiatric disability still increased from 0.10‰ in 1987 to 0.77‰ in 2006. In both surveys, males, rural residents, Han nationality and 4–6 people in the household accounted for the majority proportion of children (Table 3).

We did not present all childhood mental disorders, such as autism, depression, disruptive behavior and so on, in the study. For example, autism was not included as an individual type of psychiatric disability in 1987 survey, but as an individual type of psychiatric disability in 2006 survey. Therefore, these above disorders were classified as others for analysis. The distributions of schizophrenia, schizotypal and delusional disorders were significantly different between the 1987 and 2006 surveys (Table 4). No differences were observed between the two surveys for other types of psychiatric disability.

Logistic analyses showed material difference in the association with demographical characteristics between the 1987 and 2006 surveys (Table 5). Age was the most important predictor of psychiatric disability. Compared with children aged 6–14 years, the probability of having a psychiatric disability increased 31% in children aged 4–5 years and decreased 41% in those aged 0–3 years in the 2006 survey; however, we did not observe the same associations in the 1987 survey. After combining these two surveys, children aged 0–3 years continued to present significantly lower ORs for psychiatric disability, and marginally increased 21% ORs for psychiatric disability were present for the 4–5 year-old age group.

There were various associations between the different survey years investigated. For example, household size showed higher OR for psychiatric disability in 2006, but we did not find the same increasing trend in 1987. Additionally, children living in rural areas were more likely to have a psychiatric disability than those living in urban areas. Otherwise, we noticed that male and ethnic minority children were exposed to environments with high risk of psychiatric disability.

## 4. Discussion

Using detailed personal interviews and professional psychiatric health examinations from nationally representative samples in 1987 and 2006, we obtained valuable data on psychiatric disability among Chinese children. Whether taking epilepsy into consideration or not, the results suggested that age-standardized point prevalence of psychiatric disability in children sharply increased from 1987 to 2006. Moreover, socioeconomic factors, such as gender, residence area and ethnicity and so on, were associated with psychiatric disability risk.

In this study, the prevalence of psychiatric disability was strikingly low compared with other studies [8,9]. Costello et al. suggested that the median prevalence of psychiatric disorders was 12% among impaired child and adolescents, although the range of this prevalence was broad [27]. The main reason for the prevalence gap between our study and others’ results was that a more narrow definition of disability, which was confirmed by physician examination, was used in both surveys [17]. Diagnostic interviews might also contribute to the prevalence gap. Additionally, there was no single best way to identify child psychiatric disorders [27]. Although the cur­rent findings might underestimate the prevalence of child psychiatric disability, the age-standardized prevalence of psychiatric disability in children still increased nearly six-fold in 20 years. Possible reason was that there is no national mental health law in China, and China has not yet given its mental health service system sufficient priority [28,29]. Currently, China lacks qualified mental health professionals [29], and there are only 1.3 psychiatrists and 2.1 psychiatric nurses per 100,000 population in China [30].

Children may suffer from a number of psychiatric and behavioral disorders during childhood and adolescence. In the current study, we found there was a different distribution of schizophrenia between the 1987 and 2006 surveys, and the number of schizophrenia cases was higher in 2006 than in 1987. A possible reason is that development of schizophrenia is increased by prenatal and perinatal events including maternal influenza, rubella, malnutrition, diabetes mellitus, and smoking during pregnancy; it is also increased by obstetric complications [31]. China is facing more serious obstetric complications issue [32]. Therefore, obstetric complications might cause the increase in schizophrenia among Chinese children.

Children of minority ethnicity may be at increased risk of psychiatric disability as the result of a number of factors including genetics, exposure to environmental and infectious agents, and social conditions such as differential experiences of discrimination and exclusion [1]. Although minority ethnicity was not an increased risk factor for psychiatric disability in 1987, it was a significant risk factor in 2006 or when these two surveys were combined. Compared with children of Han ethnicity, those of minority ethnicity in China are more likely to live in poverty with less access to social security and education, especially those with mental disabilities [33]. Moreover, the place of residence might reflect important differences in exposure to environmental contaminants, access to nutritious food, social or other risk factors for children with psychiatric disability [1]. In the current study, we observed that rural areas had higher ORs for child psychiatric disability in both surveys. The possible reason is that those living in Chinese rural areas experience more difficulty accessing nutritious food or are more likely to suffer exposure to environmental risks compared with those living in urban areas.

With increasing household size, we found a negative association with child psychiatric disability in 1987, although it was not statistically significant. However, we found a significant positive association with child psychiatric disability in 2006. These unique and interesting associations have rarely been observed in previous studies among children. A review of the literature showed that large household size correlates with factors such as low income, poor parental behaviors, inadequate parental supervision, and a lack of attention, affection and family interaction [34]. As for different child psychiatric disability associations, it is possible that a large family is overburdened and therefore unable to provide enough support for the children; this is especially true for the 2006 survey [35]. In contrast, household size may not have been so closely linked with socioeconomic context in the 1980s. Moreover, it must be kept in mind that the children surveyed in 1987 were born in the 1970s and 1980s, whereas those surveyed in 2006 were born in the 1990s and 2000s. China has undergone marked changes in family structure, and socioeconomic, political, institutional and demographic transitions during the past 30 years.

The present study also had some weaknesses. All the participants in the survey were first screened for disability and only those suspected of being disabled were examined and diagnosed. Therefore, children with psychiatric disability who were not disabled might not have been identified during the survey, which would cause the number cases of psychiatric disability was underestimated. Moreover, the international classification of impairments, disabilities, and handicaps was used in the 1987 survey [18], and the international classification of functioning, disability, and health was used to classify disability in the 2006 survey [19]. Both surveys used the same Chinese word “Canji”, which means both handicap and disability, and helps to keep the consistency of the definition used in both surveys. Although there were some differences in screening methods, diagnostic methods and the classification of psychiatric between 1987 and 2006, they were comparable and presented good reliability and validity [23,24,25], it should be cautious for future studies.

## 5. Conclusions

China is undergoing social and economic changes. Our results will be of benefit for understanding psychiatric disability and 20-year change in children under 14 years old. The government should also continue to improve social security and health care systems across the country, especially for minority ethnicity children and children who lived in the rural. Additionally, China faces a great challenge to its mental health system. The government should adjust its current mental health system policies and implementation strategies.

## Figures and Tables

**Table 1:** Screening questions for mental disability in different age groups in 1987.

Age Group (Years)	Screening Questions
0–14	Are you or your family members having abnormal behavior or speaking? Or do you have any suspicious mind? Or do you have aural/visual hallucination? Or can you not control your moods? Or does your personality aggressively change? Or are you addicted to alcohol or drugs? And does your child always be alone and not play with others? Or does your child always escape school?
15+	Are you or your family members having abnormal behavior or speaking? Or do you have any suspicious mind? Or do you have aural/visual hallucination? Or can you not control your moods? Or does your personality aggressively change? Or are you addicted to alcohol or drugs?

**Table 2:** Screening questions for mental disability in different age groups in 2006.

Age Group (Years)	Screening Questions
0–6	Does your child lack of eye contact? Or does your child hear but no response? Or does your child always be alone and not play with others? Or does your child loss interests in the environment or always have stereotyped, repetitive behaviour? Or does your child have verbal and non-verbal communication disorders?
7–17	Are you or your family members forgetful? Or do you have difficulty concentrating? Or can you not control your moods? Or do you have strange behavior that is out of the ordinary? Or are you addicted to alcohol or drugs? Or does your child lack of eye contact? Or does your child hear but no response? Or does your child always be alone and not play with others? Or does your child loss interests in the environment or always have stereotyped, repetitive behaviour? Or does your child have verbal and non-verbal communication disorders?
18+	Are you or your family members forgetful? Or do you have difficulty concentrating? Or can you not control your moods? Or do you have strange behavior that is out of the ordinary? Or are you addicted to alcohol or drugs?

**Table 3:** Characteristics of psychiatric disability and associations with demographic in Chinese children *.

Variable	1987	2006
	Sample Size and Proportion *n* (%)	Sample Size and Proportion *n* (%)
Age group (years)		
6–14	280,300 (60.9)	313,466 (65.4)
4–5	62,843 (13.7)	56,619 (11.8)
0–3	117,261 (25.5)	109,496 (22.8)
*p* (1987 vs. 2006)	<0.01	
Gender		
Male	238,535 (51.8)	257,719 (53.7)
Female	221,869 (48.2)	221,862 (46.3)
*p* (1987 vs. 2006)	<0.01	
Residence		
Urban	115,150 (25.0)	129,757 (27.1)
Rural	345,254 (75.0)	349,824 (72.9)
*p* (1987 vs. 2006)	<0.01	
Ethnicity		
Han	407,558 (88.5)	406,013 (84.7)
Others	52,846 (11.5)	73,568 (15.3)
*p* (1987 vs. 2006)	<0.01	
Household size		
1–3	60,688 (13.2)	140,518 (29.3)
4–6	306,572 (66.6)	304,825 (63.6)
7–9	93,144 (20.2)	34,238 (7.1)
*p* (1987 vs. 2006)	<0.01	
Total	460,404	479,581

* Chi-square test for difference proportion of categorical variables.

**Table 4:** Distribution of four most cases of psychiatric disability types in Chinese children psychiatric disability population by age groups *.

Selected Types of Psychiatric Disability, *n*	1987			2006		
	0–3	4–5	6–14	0–3	4–5	6–14
Organic mental disorders						
Male	1	1	7	6	5	37
Female	0	0	1	5	10	30
*p* (1987 vs. 2006)	0.10					
Schizophrenia, schizotypal and delusional disorders						
Male	0	0	1	0	1	17
Female	0	3	0	0	1	8
*p* (1987 vs. 2006)	<0.01					
Epilepsy						
Male	0	2	19	7	9	76
Female	0	2	14	5	5	53
*p* (1987 vs. 2006)	0.87					
Others						
Male	1	0	2	12	13	46
Female	0	0	3	14	10	33
*p* (1987 vs. 2006)	0.83					

* Fisher’s exact chi-square test for different distribution of selected types of psychiatric disability.

**Table 5:** Associations of psychiatric disability in Chinese children.

	Reference		OR (95% CI)
***1987 and 2006 year***			
Investigation year	1987	2006	**6.35 (4.88–8.27)**
Age group (years)	6–14	4–5	1.21 (0.97–1.51)
		0–3	**0.52 (0.41–0.65)**
Gender	Male	Female	**0.77 (0.65–0.91)**
Residence	Urban	Rural	0.93 (0.77–1.13)
Ethnicity	Han	Others	**1.61 (1.32–1.95)**
Household size	1–3	4–6	**1.27 (1.05–1.57)**
		7–9	**1.42 (1.04–1.94)**
***1987***			
Age group (years)	6–14	4–5	0.68 (0.35–1.32)
		0–3	**0.11 (0.03–0.35)**
Gender	Male	Female	0.84 (0.53–1.31)
Residence	Urban	Rural	**2.01 (1.06–3.84)**
Ethnicity	Han	Others	1.44 (0.77–2.68)
Household size	1–3	4–6	0.90 (0.42–1.91)
		7–9	0.65 (0.26–1.61)
***2006***			
Age group (years)	6–14	4–5	**1.31 (1.04–1.66)**
		0–3	**0.59 (0.46–0.76)**
Gender	Male	Female	**0.77 (0.64–0.91)**
Residence	Urban	Rural	1.18 (0.95–1.45)
Ethnicity	Han	Others	**1.61 (1.31–1.98)**
Household size	1–3	4–6	**1.25 (1.01–1.54)**
		7–9	**1.53 (1.09–2.14)**

Abbreviations: CI = confidence interval; OR = odds ratio; Bold writing: *p* < 0.05.
